# Designing Versatile Superhydrophilic Structures via an Alginate-Based Hydrophilic Plasticene

**DOI:** 10.3390/mi14050962

**Published:** 2023-04-28

**Authors:** Wenbo Shi, Haoyu Bai, Yaru Tian, Xinsheng Wang, Zhe Li, Xuanbo Zhu, Ye Tian, Moyuan Cao

**Affiliations:** 1 State Key Laboratory of Chemical Engineering, School of Chemical Engineering and Technology, Tianjin University, Tianjin 300072, China; ryan_2020207337@tju.edu.cn (W.S.); bhyy@tju.edu.cn (H.B.); hblz@tju.edu.cn (Z.L.); 2 School of Materials Science and Engineering, Smart Sensing Interdisciplinary Science Center, Nankai University, Tianjin 300350, China; 2120221331@mail.nankai.edu.cn (Y.T.); wangxs1996@outlook.com (X.W.); 3 Haihe Laboratory of Sustainable Chemical Transformations, Tianjin 300192, China; 4 National and Local Joint Engineering Laboratory for Synthetic Technology of High-Performance Polymer, College of Chemistry, Jilin University, Changchun 130012, China; 5 College of Medicine and Biological Information Engineering, Northeastern University, Shenyang 110819, China

**Keywords:** superhydrophilic, plasticene, hierarchical structure, spontaneous transport, solar evaporation

## Abstract

The rational design of superhydrophilic materials with a controllable structure is a critical component in various applications, including solar steam generation, liquid spontaneous transport, etc. The arbitrary manipulation of the 2D, 3D, and hierarchical structures of superhydrophilic substrates is highly desirable for smart liquid manipulation in both research and application fields. To design versatile superhydrophilic interfaces with various structures, here we introduce a hydrophilic plasticene that possesses high flexibility, deformability, water absorption, and crosslinking capabilities. Through a pattern-pressing process with a specific template, 2D prior fast spreading of liquids at speeds up to 600 mm/s was achieved on the superhydrophilic surface with designed channels. Additionally, 3D superhydrophilic structures can be facilely designed by combining the hydrophilic plasticene with a 3D-printed template. The assembly of 3D superhydrophilic microstructure arrays were explored, providing a promising route to facilitate the continuous and spontaneous liquid transport. The further modification of superhydrophilic 3D structures with pyrrole can promote the applications of solar steam generation. The optimal evaporation rate of an as-prepared superhydrophilic evaporator reached ~1.60 kg·m^−2^·h^−1^ with a conversion efficiency of approximately 92.96%. Overall, we envision that the hydrophilic plasticene should satisfy a wide range of requirements for superhydrophilic structures and update our understanding of superhydrophilic materials in both fabrication and application.

## 1. Introduction

Interfaces possessing special functions have emerged as a powerful platform for innovating materials and devices in both scientific research and modern technology [1,2,3,4,5,6]. The extreme interaction between fluids and a superwetting interface can facilitate a series of fluid-controlling processes such as liquid-repellency [7,8], fluid separation [9], gas extraction [10], etc. In general, lotus-leaf-inspired superhydrophobic and fish-scale-inspired superhydrophilic interfaces represent typical superwettable materials. Due to the superior liquid shielding ability, the superhydrophobic materials can be applied in the field of anti-icing [11], corrosion resistance [12], self-cleaning paint [13,14], etc. On the basis of the high affinity for water, superhydrophilic materials exhibit great potential in wound dressing [15,16], oil/water separation [17], microfluidics [18,19,20], etc. The design of superwettable materials with complex and hierarchical structures should offer great opportunities to optimize the current systems relating to the multi-phase interaction.

To realize such unique properties, the micro-/nano-scale structure and the chemical composition of the surface should be cooperatively designed [21,22]. As an inspiration from nature, lotus leaves have two-tier microstructure structures covered with hydrophobic plant wax, i.e., the micro-protrusion and nanoscale roughness [23,24,25]. Interestingly, a similar structure can be found in fish scales that possesses superhydrophilic properties [26,27]. In general, the microgroove structure of a superhydrophilic surface is able to generate the capillary force for rapid liquid delivery, and the nanoscale surface roughness can trap liquid molecules for lowering the hysteresis of liquid motion. Differing with the superior water repellency of superhydrophobic interfaces, superhydrophilic surfaces show promising functions such as liquid pumping [28,29,30], anti-fouling [31,32,33], directional floatability [34], etc.

The rational design of superhydrophilic surfaces with hierarchical structures should unlock more possibilities for their development and application [35]. A Chinese Xuan paper composed of aligned nanofibers is one of the most famous superhydrophilic materials with 2D prior liquid spreading ability [36]. Inspired by the surface morphology of animal’s corneas, Miao et al. reported a liquid superspreading process based on the unique microchannel and nanofiber array. Thanks to the cooperative surface structure, the spreading velocity on such a surface is 26 times faster than the conventional superhydrophilic surface [37]. In addition to the 2D superhydrophilic surfaces, 3D superhydrophilic structures are also of great potential in liquid manipulation. With a hierarchical microchannel structure, the conical Sarracenia trichome can achieve ultrafast water harvesting with a droplet velocity of 11,738  ±  715 μm s^−1^, exhibiting a significant acceleration as compared with the smooth conical spine [38]. Through the vertical superhydrophilic, light-absorbing stick array, Li et al. realized an enhanced evaporation rate exceeding 100% solar-to-vapor conversion by harvesting both solar and environmental energy [39].

Although the superhydrophilic structure has a series of unique liquid manipulation abilities, the universal and facile strategies of design and fabrication should be further investigated to meet the requirement of hierarchical structure and complicated design. To this end, here we report a hydrophilic plasticene for designing versatile superhydrophilic interfaces. The alginate-based plasticene can be easily shaped with proper templates and rapidly solidified by calcium ions. Silica microparticles and wood microfibers acted as the fillers of this plasticene, which endowed the as-solidified superhydrophilic structure with a hydrophilic capillary structure and fire retardation. Taking the advantages of the high deformability of the plasticene, versatility of the superhydrophilic structures for liquid manipulation can be designed such as the 2D-patterned paper, 3D microstructure arrays, complex and hierarchical channels, etc. Furthermore, the superhydrophilic structure is able to be modified with pyrrole molecules, resulting in a light-absorbing substrate for constructing the solar steam generator with promising performance. We envision that this hydrophilic plasticene can be a good candidate for the preparation of superhydrophilic structures, which provides a useful platform for incorporating superhydrophilic interfaces into different application systems.

## 2. Materials and Methods

Materials and Characterization: In this study, wood fiber (FB-300) and SiO_2_ particles were obtained from Shanghai Yingjia Industrial Development Co., Ltd. (Shanghai, China) and Dongwan Xinweijin Industrial Co., Ltd. (Dongwan, China), respectively. Sodium alginate, calcium chloride, hydrochloric acid, sodium hydroxide, 5% H_2_O_2_ solution, ammonium persulfate, and Pyrrole solution were purchased from Shanghai Macklin Chemical Co., Ltd. (Shanghai, China) without any further treatment. The surface morphology of the black and white hydrophilic plasticene, as well as different parts of the hydrophilic plasticene cube after six minutes of combustion under a butane flame, was analyzed using a scanning electron microscope (Apreo S LoVac, FEI Co., Ltd., Hillsboro, Oregon, USA). Tailored molds were printed using a 3D printer (Formlabs, Form 2), and water contact angles of the surfaces were measured using an SDC-200 geometer (SFMIT Co., Changzhou, China) with a 10 μL droplet. The detailed droplet growing processes were recorded using an industrial microscope (GP-650S, Gaopin Co., Kunshan, China), while the processes of 2D prior spreading on the hydrophilic plasticene were recorded using a high-speed camera (5KF10, Revealer, Hefei, China). A syringe pump (SP-2000, Annol Co., Ningbo, China) was utilized to discharge water droplets at a constant flow rate of 20 mL min^−1^. Solar steam generation was measured using a solar simulator (SOLAR-500, NBeT, Beijing, China) with an AM 1.5 optical filter. The light intensity was regulated to 1 sun (1000 W m^−2^) with a solar power meter (TM-207, TENMARS, Taipei, Taiwan, China) and the solar absorption at 200–2500 nm was measured using a UV–VIS-NIR spectrophotometer (PE Lambda 750, Perkin Elmer, USA) equipped with a 150 mm integrating sphere. A thermal infrared camera (E6, FLIR, Wilsonville, Oregon, USA) was utilized to capture the thermal images, while the mechanical properties of the hydrophilic plasticene were analyzed using an electromechanical universal testing machine (CMT8502, MTS SYSTEMS CHINA Co., Ltd., Shanghai, China). 

Preparation of the hydrophilic plasticene with Complex Structure: To create the hydrophilic plasticene material, a mixture of wood fibers, SiO_2_ particles, and sodium alginate in a mass ratio of 8:16:1 was prepared and pressed into various shapes using 3D-printed molds. The resulting samples were then immersed in a 10 wt% CaCl_2_ solution for 1 h and washed to remove excess particles before being air-dried. To fabricate the black hydrophilic plasticene, the hydrophilic plasticene was first crosslinked and dried. It was then wetted with a dropwise addition of 1 mL of an aqueous ammonium persulfate solution (10 wt%) and dried again at 50 °C for 1 h. A 5% solution of pyrrole ethanol was then dripped onto the treated hydrophilic plasticene and dried for an additional hour at 50 °C, resulting in the formation of the black hydrophilic plasticene material.

## 3. Results and Discussion

### 3.1. Design and Fabrication of Hydrophilic Plasticene

**Figure 1:**
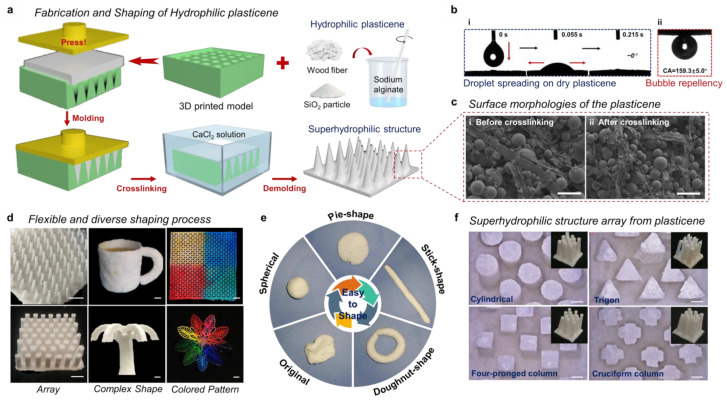
The design and the properties of hydrophilic plasticene. (**a**) Fabrication and shaping of hydrophilic plasticene. (**b**) Hydrophilic properties and bubble repellency of plasticene. (**i**) Water droplets (10 µL) spread rapidly on the surface of plasticene. (**ii**) Underwater bubble (4 µL) contact angle of plasticene. (CA = 159.3 ± 5.0°) (**c**) Surface morphologies of the plasticene. The SEM images show the morphologies of the plasticene (**i**) before and (**ii**) after crosslinking. The scale bar is 100 μm. (**d**) Flexible and diverse shaping process. Three typical examples are shown: array, complex shape, colored pattern. The scale bar is 5 mm. (**e**) Hydrophilic plasticene shaping process. (**f**) Superhydrophilic structure array from plasticene. The scale bar is 1 mm.

To develop an economically viable and practical hydrophilic plasticene, we employed natural materials that are abundant, cost-effective, and environmentally friendly. The manufacturing process also excluded the use of toxic reagents or solvents, which makes it scalable and convenient. As illustrated in Figure 1a, the hydrophilic plasticene was prepared by blending wood fibers (50 μm diameter), SiO_2_ particles (40 μm particle size), and an alginate-based polymer. This mixture was then pressed into a 3D printing mold to form the original hydrophilic plasticene. The hydrophilic slurry composed of sodium alginate, wood fibers, and SiO_2_ particles was filled into the internal or surface channels of the 3D printing mold with force, as shown in Figure S1 and Movie S1. Finally, the hydrophilic slurry and the 3D printing mold were crosslinked in a CaCl_2_ solution for 1 h, and the untethered particles were removed using water flushing. The SEM analysis (Figure 1c) confirmed the relatively dense structure of the prepared hydrophilic plasticene, which provided sufficient mechanical strength. The hydrophilic plasticene film surface was flat, and 10 µL droplets spread quickly when it dried. Moreover, the contact angle of the bubbles (4 µL) in the underwater environment was measured to be 159.3° ± 5.0°, facilitating bubble support and thorough wetting of the droplets (Figure 1b).

The pliable nature of an unsolidified hydrophilic plasticene allows for facile shaping with external force, enabling it to adopt a wide range of forms from spherical to doughnut-shaped with ease (Figure 1e and Movie S2). By utilizing 3D printing molds, flexible and diverse shaping processes can be implemented, including the formation of arrays, complex shapes, and colored patterns (Figure 1d). With this method, the smallest feature size can be manufactured for a 2D structure with a thickness of ~0.1 mm and a 3D structure with a cone of 1 mm diameter. The cylindrical, trigon, four-pronged column, and cruciform column arrays are shown in Figure 1f. The hydrophilic plasticene material exhibits exceptional functionality, including liquid absorption and underwater bubble repellency. For example, dendritic hydrophilic plastisol demonstrates the capability of grabbing and releasing air bubbles in an underwater environment (Figure S2 and Movie S3). Moreover, the assembly ability of plasticene is highly promising. By employing a water welding process, hydrophilic plasticene can assemble complex structures prior to crosslinking (Figure S3 and Movie S4). The water welding process occurs when the water droplet drips on the alginate-based plasticene surface, which is dried and molded without crosslinking. The wetted, uncrosslinked surface becomes sticky due to the dissolving process and possesses remarkable adhesion ability. By attaching another dry, alginate-based plasticene on the adhesive surface, the two surfaces will self-weld and integrate into a cohesive plasticene. However, the welding process will be disabled once the hydrophilic plasticene is crosslinked by calcium ions. The flower-shaped, superhydrophilic kirigami mode of plasticene capitalizes on its fluid-wicking ability, enabling liquid transport from the end of the petal to the center of the flower (Figure S4 and Movie S5). Furthermore, solidified plasticene maintains its morphology and internal structure under acidic, alkaline, and oxidizing conditions, indicating its sufficient durability with respect to liquid interactions (Movie S5). The flame resistance of plasticene has also been validated. The combustion behavior of hydrophilic plasticene can be analyzed via SEM observation of different parts of hydrophilic plasticene burning via butane flame (Figure S6 and Movie S6). Additionally, the water permeability (Figure S7) and mechanical properties (Figure S8) of hydrophilic plasticenes were also investigated. For dry samples, the material exhibits a maximum strain of ε_max_ = 5.33% and a tensile strength of σ_max_ = 24.81 MPa. In contrast, for wetted samples, the material exhibits a maximum strain of ε_max_ = 28.21% and a tensile strength of σ_max_ = 3.24 MPa. The results demonstrate that hydrophilic plasticenes are brittle in their dry state and flexible in their wet state.

Sodium alginate and wood fiber are widely available natural resources, while SiO_2_ particles are effective hydrophilic materials that can enhance the density of hydrophilic plasticene by filling the voids. By employing a well-considered design and fabrication approach that enables the creation of various patterns, shapes, and structures, the resultant hydrophilic plasticene demonstrates superior liquid spreading in two dimensions, which is promising in continuous liquid transport and for efficient interfacial solar evaporation capabilities.

### 3.2. 2D Prior Spreading on Hydrophilic Plasticene

**Figure 2:**
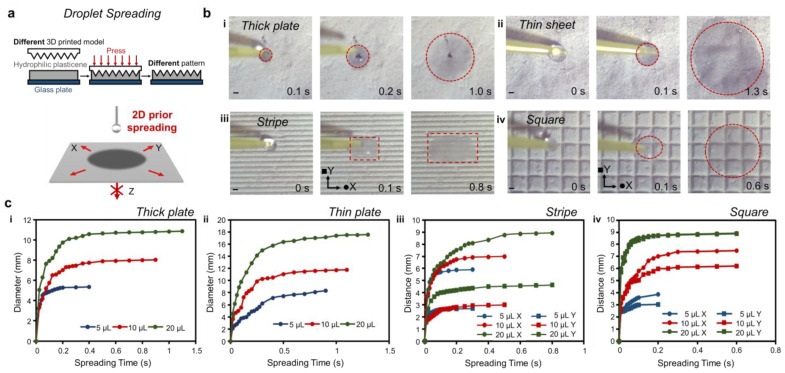
2D prior spreading on hydrophilic plasticene. (**a**) Schematic diagram of droplet spreading. Hydrophilic playdough can be imprinted with different patterns according to different 3d printing models. Fast spreading on 2D plate (X and Y) is favored, and transportation along the normal direction (Z) is restrained. (**b**) Spreading of water (20 µL) on (**i**) thick plate, (**ii**) thin plate, (**iii**) stripe, and (**iv**) square is finished in a short time. The scale bar is 1 mm. (**c**) Function between the spreading time and diameter or distance.

Achieving controllable liquid spreading is crucial for many applications in interfacial materials. In this study, we investigated the 2D prior spreading on a superhydrophilic sheet made of plasticene with varying patterns. Sheets of hydrophilic plasticene with different thicknesses and patterns were prepared using 3D printing molds (Figure 2a). To study the wetting behavior of droplets on hydrophilic plasticene, we recorded the wetting process of droplets of different volumes (5, 10, 20 μL) using a high-speed camera and extracted the typical frames (Figure 2b and Movie S7). The water spot diameter or distance was then measured against spreading time, which generated the curve depicted in Figure 2c. The spreading speed of the droplet against spreading time is represented in the curve shown in Figure S9. The entire spreading process of the droplet was completed within a few seconds, and the spot size increased rapidly. The curves displayed a sawtooth tendency, which indicates a decrease and increase in the spreading speed repeatedly until the spreading was complete. The thicker plates, stripes, and squares spread liquid faster than the thin sheets, and the liquid did not penetrate the interface. It is worth noting that thick plates, stripes, and squares are 10–20 times thicker than thin sheets, which are only about 0.1 mm thick. The spreading of water on these surfaces is rapid. (For a droplet volume of 20 μL, the complete spreading time on a thick plate, thin sheet, stripe, and square is 1.0, 1.3, 0.6, and 0.6 s, respectively.) The unique wettability of hydrophilic plasticene is characterized by 2D prior spreading, indicating that liquid spreading is limited to the X and Y directions but not the Z direction (Figure 2a).

Through comparative experiments involving thick plates and thin sheets, it was observed that, for the same volume of droplets deposited on unpatterned superhydrophilic surfaces, the water spot formed on the thin sheet was approximately twice as large as that formed on the thick plate. However, the spreading speed on the thin sheet was even faster than that on the thick plate. Notably, thinner hydrophilic plasticene thicknesses were found to facilitate the 2D spreading of droplets, whereas thicker thicknesses improved resistance to permeation, owing to the multilayer diffusion mechanism that necessitates water to overcome more obstacles to penetrate.

Embossing a striped pattern onto a flat sheet was found to encourage liquid on the stripes to spread preferentially along the direction of the stripes (X direction) upon contact. For droplet spreading on the stripes, the spreading speed in the X direction was found to be 1.6–2.2 times higher than that in the Y direction. This finding suggests that the channels generated by the imprint on hydrophilic plasticene have a guiding and facilitating effect on liquid spreading. In an effort to extend the channel advantage, a square pattern with channels in both the X and Y directions was designed. The droplet spreading speed on this square pattern was the fastest, reaching up to 600 mm/s, with spreading speed and spreading time in the X direction being similar to those in the Y direction. Ultimately, the surface-oriented patterned channels were found to contribute to monolayer spreading of the water droplets, a unique wettability attributed to the layered micro/nanostructure of the surface, which facilitates multilayer spreading.

### 3.3. Liquid Transport of 3D Superhydrophilic Structure

Hydrophilic plasticene, owing to its arbitrary moldability, offers a promising avenue for continuous liquid transport. The hydrophilic nature of the material and the 3D-embossed complex structure makes it an ideal candidate for liquid-conducting devices. A plethora of attempts have been undertaken to enhance the speed and efficiency of liquid transport, leading to the discovery of some fascinating phenomena. In this regard, five distinct types of single-row, cylindrical arrays were fabricated by pressing hydrophilic plasticene into 3D-printed molds, which served as an experimental platform to study the liquid transport process. The five types of cylindrical arrays include the following: sample Ⅰ, which is a solid pillar with a solid channel (the diameter of the pillar is 1.5 mm and the height is 5 mm); sample Ⅱ, which is a solid pillar with a hollow channel (the width and depth of the channel are 2 mm); sample Ⅲ, which is a half-solid pillar with a hollow channel (the diameter of the hollow part of pillar is 1 mm); sample Ⅳ, which is a hollow pillar with a hollow channel; and sample Ⅴ, which is an open pillar with a hollow channel.

**Figure 3:**
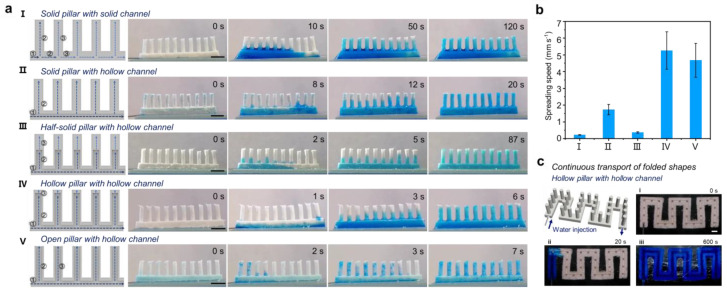
Liquid transport on different morphological single-row pillar hydrophilic plasticene. (**a**) Liquid transport process of five single-row pillar hydrophilic plasticene. Sample Ⅰ is a solid pillar with a solid channel. Sample Ⅱ is a solid pillar with a hollow channel. Sample Ⅲ is a half-solid pillar with a hollow channel. Sample Ⅳ is a hollow pillar with a hollow channel. Sample Ⅴ is an open pillar with a hollow channel. The scale bar is 5 mm. (**b**) Comparison of liquid spreading rate of hydrophilic plasticene for five single-row pillars. (**c**) Continuous transport of folded shapes of hollow pillar with hollow channels. The photos show the bottom view. (**i**) Water is injected from the inlet, (**ii**) can be transported to the outlet, and (**iii**) flows out. The scale bar is 5 mm.

To investigate the fluid transport in different types of 3D superhydrophilic arrays, a 200 µL fluid injection at a speed of 2 mL/min was performed from the bottom of each 3D sample. The experiment was recorded using Figure 3a and Movie S8. Liquid flow on sample Ⅰ tended to accumulate at the inlet side due to the large influx of liquid and the saturation of liquid absorption. However, the liquid was eventually transported spontaneously by being absorbed by the dry parts. Sample Ⅱ showed faster transport due to the penetration of both sides of the channel, and the reduction of overall transport time due to the opening of the trench. Sample Ⅲ, made of a semi-solid column based on sample Ⅱ, exhibited slow pumping of hydrophilic solid pillar, which resulted in slow liquid spreading velocity. Sample Ⅳ showed a drastic reduction in liquid transport time due to the increase in cavities in the structure and the decrease in hydrophilic plasticene. Sample Ⅴ showed similar liquid transport time to sample Ⅳ, but the liquid transport mode was different due to the open top of the pillar. The average speed of complete transport is shown in Figure 3b. Samples Ⅳ and Ⅴ demonstrated fast fluid transport rates, whereas sample Ⅰ had the slowest. The liquid was transported on sample Ⅴ by first spreading the hollow channel and then filling the hollow pillar at the same time. However, for sample Ⅳ, the liquid was transported from the inlet side down the hollow channel to fill the hollow pillar sequentially. Additionally, fluid transport was observed to occur continuously on folded shapes, further demonstrating the potential of these 3D hydrophilic plasticene structures for practical applications in liquid transport systems.

### 3.4. The Evaporation Performance of Hydrophilic Plasticene

**Figure 4:**
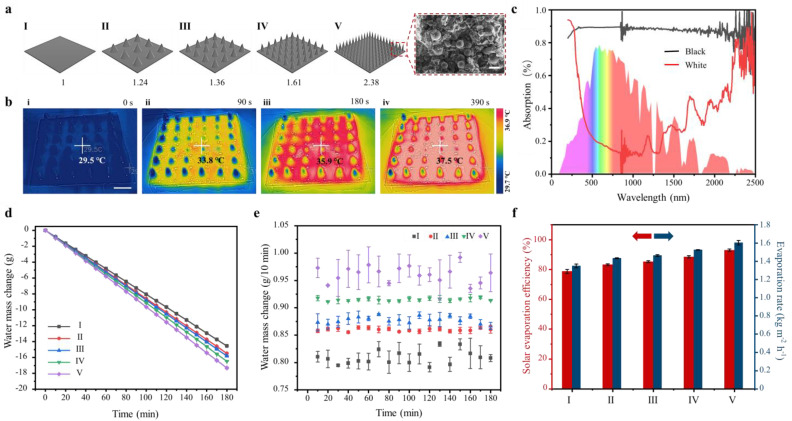
The in-lab solar evaporation performance of black hydrophilic plasticene. (**a**) Five different sizes of cone arrays prepared from black hydrophilic plastic were applied for solar evaporation. Sample Ⅰ is a 6 cm × 6 cm square with an area of 3600 cm^2^; if its area is considered as “unit 1”, then the other arrays of the exposed area can be expressed as such. Sample Ⅱ = 1.24, Ⅲ = 1.36, Ⅳ = 1.61, Ⅴ = 2.38. The SEM images show the upper surface of black hydrophilic plasticene covered with polypyrrole. The scale bar is 50 μm. (**b**) The increase process of the surface temperature of Ⅳ during the solar evaporation process by using thermal images captured by a thermal infrared camera. The scale bar is 1 cm. (**c**) Ultraviolet–visible-near-infrared (UV–VIS-NIR) spectra of hydrophilic plasticene between 200–2500 nm. The light absorption of black hydrophilic plasticene and white hydrophilic plasticene are shown. The water mass change (**d**,**e**), evaporation rate, and solar evaporation efficiency (**f**) during 180 min indoor solar evaporation under 1 sun (1 kW m^−2^).

The 3D superhydrophilic structure has shown great potential in facilitating liquid evaporation processes due to its inherent liquid-pumping and spreading ability. To enhance the evaporation efficiency, we aimed to increase the actual air–liquid interface area per unit area by introducing micro-cones onto the superhydrophilic sheet using a template pressing method (Figure 4a and Figure S10). To apply hydrophilic plasticene in solar evaporation, we fabricated a black light-absorbing layer on the surface of the material through a chemical reagent reaction. The black layer, which mainly consisted of polypyrrole, provided only a staining effect on hydrophilic plasticene and did not affect the internal structure of the material, as evidenced by the SEM image (Figure 4a). Under one sunlight irradiation, the surface temperature of hydrophilic plasticene with the black layer increased by 4 °C within 90 s, with the maximum temperature reaching 37.5 °C and remaining stable around the peak (Figure 4b). This enhanced interfacial temperature is attributed to the high absorption of solar irradiation by the black layer (Figure 4c). Our findings demonstrate the potential of the 3D superhydrophilic structure in enhancing the performance of hydrophilic plasticene in solar evaporation applications.

In order to evaluate the evaporation performance of hydrophilic plasticene, we conducted laboratory experiments using simulated solar light with a density of 1000 W/m^2^ to accelerate the interfacial evaporation. The evaporation capacity was assessed quantitatively by monitoring the water mass loss (Figure 4d) over a period of 3 h, with data recorded every 10 min (Figure 4e). The recorded data indicated a linear decline in water mass over time during daylight evaporation, indicating the steady state of evaporation. Notably, the mass of water reduction per 10 min was found to be proportional to the exposed area of each sample, with a larger exposed area resulting in a greater reduction in water mass. Importantly, the relationship between the mass of water reduction and the exposed area was found to be consistent across all samples (Ⅰ–Ⅴ). This conclusion was supported by the result of natural evaporation of samples Ⅰ–Ⅴ, which were made from hydrophilic plasticene without a black light-absorbing layer and conducted in a room without sunlight irradiation (Figure S11).

The evaporation performance of the black light-absorbing hydrophilic plasticene was evaluated under one sun irradiation. The water mass change was monitored every 30 min, and the evaporation rate of black Ⅴ was found to be ~1.60 kg·m^−2^·h^−1^ with a conversion efficiency of approximately 92.96%. In contrast, the evaporation rate of black Ⅰ was only ~1.35 kg·m^−2^·h^−1^ with a conversion efficiency of approximately 78.71%. These results clearly indicate that the evaporation efficiency of hydrophilic plasticene can be enhanced by increasing the exposed area.

Moreover, this advantage will be further magnified under daylight evaporation, making it highly suitable for similar evaporation scenarios. Furthermore, the approach of shaping hydrophilic plasticene can be extended to other shape arrays (Figure S12), paving the way for novel methods to improve solar evaporation systems.

## 4. Conclusions

The development of versatile and simple superhydrophilic structures can advance the current state of superwetting interfaces. This study reports the use of hydrophilic plasticene to fabricate 2D-patterned papers, 3D microstructure arrays, and complex layered channels. The establishment of surface-oriented patterned channels enables monolayer spreading of water droplets, and this unique wettability is attributed to the layered micro/nanostructure, which facilitates multilayer spreading. The square-patterned surface exhibits the fastest spreading speed of droplets, up to 600 mm/s. Different 3D microstructure arrays were tested for continuous fluid transport, and structures with hollow pillars and channels were found to contribute to efficient liquid transport. The array design of hydrophilic plasticene inspires a method to improve the efficiency of solar evaporation. The achieved evaporation rate is ~1.60 kg·m^−2^·h^−1^ with a conversion efficiency of approximately 92.96%. The use of hydrophilic plasticene expands the research scope of superhydrophilic interfaces and solar evaporation to meet the needs of various fields, such as wound dressing, oil/water separation, and microfluidics.

## Data Availability

Not applicable.

## Supplementary Materials

The following supporting information can be downloaded at: https://www.mdpi.com/article/10.3390/mi14050962/s1. Figure S1: shaping process of hydrophilic plasticene; Figure S2: gripping and releasing process of bubbles by tree-like hydrophilic plasticene in underwater environment; Figure S3: water welding process of hydrophilic plasticene; Figure S4: colorful liquid transport process of flower-shaped hydrophilic plasticene; Figure S5: alkali, acid and oxidation resistance of hydrophilic plasticene; Figure S6: superior fire retardancy of hydrophilic plasticene; Figure S7: water permeability of hydrophilic plasticene; Figure S8: mechanical properties of hydrophilic plasticene; Figure S9: relationship between spreading velocity and time of hydrophilic plasticene; Figure S10: photographs of the solar evaporation ability test of hydrophilic plasticene in laboratory; Figure S11: the in-lab evaporation performance of hydrophilic plasticene (without solar light); Figure S12: the in-lab evaporation performance of with or without cylindrical whole row of shaped hydrophilic plasticene and 30 μL water (without solar light); Movie S1: the 3D shaping process of hydrophilic plasticene (2× speed); Movie S2: the deformability of hydrophilic plasticene (8 × speed); Movie S3: grasping and releasing of bubbles by the superaerophobic “tree” made of hydrophilic plasticene; Movie S4: water welding process of hydrophilic plasticene (2× speed); Movie S5: the rapid dying process of flower-like superhydrophilic pattern (2× speed); Movie S6: the fire retardancy of hydrophilic plasticene (40× speed); Movie S7: 2D prior liquid spreading process on superhydrophilic sheets with different structures (0.1× speed); Movie S8: liquid transport on 3D superhydrophilic pillar arrays with varying infrastructure.

## References

[1] Tian Y., Su B., Jiang L. (2014). Interfacial Material System Exhibiting Superwettability. Adv. Mater..

[2] Zhang S.N., Huang J.Y., Chen Z., Lai Y.K. (2017). Bioinspired Special Wettability Surfaces: From Fundamental Research to Water Harvesting Applications. Small.

[3] Liu H., Wang Y.D., Huang J.Y., Chen Z., Chen G.Q., Lai Y.K. (2018). Bioinspired Surfaces with Superamphiphobic Properties: Concepts, Synthesis, and Applications. Adv. Funct. Mater..

[4] Wang D.H., Sun Q.Q., Hokkanen M.J., Zhang C.L., Lin F.Y., Liu Q., Zhu S.P., Zhou T.F., Chang Q., He B. (2020). Design of robust superhydrophobic surfaces. Nature.

[5] Du H., Fan J., Miao C., Gao M., Liu Y., Li D., Feng J. (2021). Recent Advances in Constructing Interfacial Active Catalysts Based on Layered Double Hydroxides and Their Catalytic Mechanisms. Trans. Tianjin Univ..

[6] Chang W., Luo K., Wang P., Abdulshaheed A.A., Li C. (2023). Sustainable dropwise condensation enabled ultraefficient heat pipes. Droplet.

[7] Jokinen V., Kankuri E., Hoshian S., Franssila S., Ras R.H.A. (2018). Superhydrophobic Blood-Repellent Surfaces. Adv. Mater..

[8] Xu J., Xiu S., Lian Z., Yu H., Cao J. (2022). Bioinspired materials for droplet manipulation: Principles, methods and applications. Droplet.

[9] Long Z.Y., Zhao Y.Y., Zhang C.H., Zhang Y.H., Yu C.M., Wu Y.C., Ma J., Cao M.Y., Jiang L. (2020). A Multi-Bioinspired Dual-Gradient Electrode for Microbubble Manipulation toward Controllable Water Splitting. Adv. Mater..

[10] Wang X.S., Bai H.Y., Yang J.R., Li Z., Wu Y.C., Yu C.M., Jiang L., Cao M.Y. (2021). Designing Flexible but Tough Slippery Track for Underwater Gas Manipulation. Small.

[11] Li X., Li X., Mu Z., Li Y., Feng F. (2023). An Experimental Study on Biochar/Polypyrrole Coating for Blade Anti-Icing of Wind Turbines. Coatings.

[12] Darband G.B., Aliofkhazraei M., Khorsand S., Sokhanvar S., Kaboli A. (2020). Science and Engineering of Superhydrophobic Surfaces: Review of Corrosion Resistance, Chemical and Mechanical Stability. Arab. J. Chem..

[13] Xie H., Wei J.F., Duan S.Y., Zhu Q., Yang Y.F., Chen K., Zhang J.J., Li L.X., Zhang J.P. (2022). Non-fluorinated and durable photothermal superhydrophobic coatings based on attapulgite nanorods for efficient anti-icing and deicing. Chem. Eng. J..

[14] Su W.B., Lu X.Y., Shu Y.X., Liu X.S., Gao W., Yao J.J., Niu Z., Xie Y.L. (2023). Robust, Superhydrophobic Aluminum Fins with Excellent Mechanical Durability and Self-Cleaning Ability. Micromachines.

[15] Chen S.L., Li S.Y., Ye Z.P., Zhang Y.F., Gao S.D., Rong H., Zhang J.H., Deng L.D., Dong A.J. (2022). Superhydrophobic and superhydrophilic polyurethane sponge for wound healing. Chem. Eng. J..

[16] Shi L.X., Liu X., Wang W.S., Jiang L., Wang S.T. (2019). A Self-Pumping Dressing for Draining Excessive Biofluid around Wounds. Adv. Mater..

[17] Wang M., Tsai H.S., Zhang C.F., Wang C.Y., Ho S.H. (2022). Effective purification of oily wastewater using lignocellulosic biomass: A review. Chin. Chem. Lett..

[18] Shi Y.X., Ye P., Wang C., Yang K.J., Guo J.H. (2022). Surface Hydrophilic Modification for Chip of Centrifugal Microfluidic Immunoassay System. Micromachines.

[19] Zhang Y.Y., Huang Z.D., Cai Z.R., Ye Y.Q., Li Z., Qin F.F., Xiao J.F., Zhang D.X., Guo Q.Q., Song Y.L. (2021). Magnetic-actuated “capillary container” for versatile three-dimensional fluid interface manipulation. Sci. Adv..

[20] Li Z., Huang Z.D., Cai Z.R., Li H.Z., Li A., Qiao Y.L., Yang J., Song Y.L. (2021). Vapor-Induced Liquid Collection and Microfluidics on Superlyophilic Substrates. ACS Appl. Mater. Interfaces.

[21] Wang Y.K., Liu Y.P., Li J., Chen L.W., Huang S.L., Tian X.L. (2020). Fast self-healing superhydrophobic surfaces enabled by biomimetic wax regeneration. Chem. Eng. J..

[22] Li Z., Cao M.Y., Li P., Zhao Y.Y., Bai H.Y., Wu Y.C., Jiang L. (2019). Surface-Embedding of Functional Micro-/Nanoparticles for Achieving Versatile Superhydrophobic Interfaces. Matter.

[23] Feng L., Li S.H., Li Y.S., Li H.J., Zhang L.J., Zhai J., Song Y.L., Liu B.Q., Jiang L., Zhu D.B. (2002). Super-hydrophobic surfaces: From natural to artificial. Adv. Mater..

[24] Groten J., Ruhe J. (2013). Surfaces with Combined Microscale and Nanoscale Structures: A Route to Mechanically Stable Superhydrophobic Surfaces?. Langmuir.

[25] Huang Z.D., Zhao Z.P., Zhao S.D., Cai X.B., Zhang Y.Y., Cai Z.R., Li H.Z., Li Z., Su M., Zhang C.Z. (2021). Lotus Metasurface for Wide-Angle Intermediate-Frequency Water-Air Acoustic Transmission. ACS Appl. Mater. Interfaces.

[26] Peng Y.B., Wen G., Gou X.L., Guo Z.G. (2018). Bioinspired fish-scale-like stainless steel surfaces with robust underwater anti-crude-oil-fouling and self-cleaning properties. Sep. Purif. Technol..

[27] Liu M.J., Wang S.T., Wei Z.X., Song Y.L., Jiang L. (2009). Bioinspired Design of a Superoleophobic and Low Adhesive Water/Solid Interface. Adv. Mater..

[28] Han J., Xing W.Q., Yan J., Wen J., Liu Y.T., Wang Y.Q., Wu Z.F., Tang L.C., Gao J.F. (2022). Stretchable and Superhydrophilic Polyaniline/Halloysite Decorated Nanofiber Composite Evaporator for High Efficiency Seawater Desalination. Adv. Fiber Mater..

[29] Yin K., Yang S., Wu J.R., Li Y.J., Chu D.K., He J., Duan J.A. (2019). Femtosecond laser induced robust Ti foam based evaporator for efficient solar desalination. J. Mater. Chem. A..

[30] Bai H.Y., Wang X.S., Li Z., Wen H.Y., Yang Y.F., Li M.Q., Cao M.Y. (2023). Improved Liquid Collection on a Dual-Asymmetric Superhydrophilic Origami. Adv. Mater..

[31] Zhang S.X., Jiang G.S., Gao S.J., Jin H.L., Zhu Y.Z., Zhang F., Jin J. (2018). Cupric Phosphate Nanosheets-Wrapped Inorganic Membranes with Superhydrophilic and Outstanding Anticrude Oil-Fouling Property for Oil/Water Separation. ACS Nano..

[32] Wen C., Guo H., Zhu Y., Bai H., Zhao W., Wang X., Yang J., Cao M., Zhang L. (2023). Fully Superhydrophilic, Self-floatable, and Multi-Contamination-Resistant Solar Steam Generator Inspired by Seaweed. Engineering.

[33] Khajouei M., Najafi M., Jafari S.A., Latifi M. (2023). Membrane Surface Modification via In Situ Grafting of GO/Pt Nanoparticles for Nitrate Removal with Anti-Biofouling Properties. Micromachines.

[34] Yang Y.F., Bai H.Y., Li M.Q., Li Z., Wang X.S., Wang P.W., Cao M.Y. (2022). An interfacial floating tumbler with a penetrable structure and Janus wettability inspired by Pistia stratiotes. Mater. Horiz..

[35] Miao W.N., Tian Y., Jiang L. (2022). Bioinspired Superspreading Surface: From Essential Mechanism to Application Published as part of the Accounts of Chemical Research special issue “Self-Assembled Nanomaterials”. Acc. Chem. Res..

[36] Zheng S., Du M., Miao W.N., Wang D.Y., Zhu Z.P., Tian Y., Jiang L. (2018). 2D Prior Spreading Inspired from Chinese Xuan Papers. Adv. Funct. Mater..

[37] Miao W.N., Zheng S., Zhou J.J., Zhang B., Fang R.C., Hao D.Z., Sun L., Wang D.Y., Zhu Z.P., Jin X. (2021). Microchannel and Nanofiber Array Morphology Enhanced Rapid Superspreading on Animals’ Corneas. Adv. Mater..

[38] Chen H.W., Ran T., Gan Y., Zhou J.J., Zhang Y., Zhang L.W., Zhang D.Y., Jiang L. (2018). Ultrafast water harvesting and transport in hierarchical microchannels. Nat. Mater..

[39] Li X.Q., Li J.L., Lu J.Y., Xu N., Chen C.L., Min X.Z., Zhu B., Li H.X., Zhou L., Zhu S.N. (2018). Enhancement of Interfacial Solar Vapor Generation by Environmental Energy. Joule.

