# Supplementary material for: Designing Versatile Superhydrophilic Structures via an Alginate-Based Hydrophilic Plasticene
Source: Micromachines (Basel). 2023 Apr 28;14(5):962. doi: 10.3390/mi14050962 (PMC10223521; doi:10.3390/mi14050962)
Supplement: Supplementary file 1 [file micromachines-14-00962-s001.zip › Supplementary Materials.pdf]

Supplementary Materials: Designing Versatile Superhydrophilic Structures via an Alginate-Based Hydrophilic Plasticene

Wenbo Shi [1,†], Haoyu Bai [1,2,3,†], Yaru Tian [2], Xinsheng Wang [2,3], Zhe Li [1,2,3], Xuanbo Zhu [4,*], Ye Tian [5,*] and Moyuan Cao [2,3,*]

1 State Key Laboratory of Chemical Engineering, School of Chemical Engineering and Technology, Tianjin University, Tianjin 300072, China; ryan_2020207337@tju.edu.cn (W.S.); bhyy@tju.edu.cn (H.B.); hblz@tju.edu.cn (Z.L.)
2 School of Materials Science and Engineering, Smart Sensing Interdisciplinary Science Center, Nankai University, Tianjin 300350, China; 2120221331@mail.nankai.edu.cn (Y.T.); ngxs1996@outlook.com (X.W.)
3 Haihe Laboratory of Sustainable Chemical Transformations, Tianjin 300192, China
4 National and Local Joint Engineering Laboratory for Synthetic Technology of High-Performance Polymer, College of Chemistry, Jilin University, Changchun 130012, China
5 College of Medicine and Biological Information Engineering, Northeastern University, Shenyang 110819, China
* Correspondence: zhuxuanbo@jlu.edu.cn (X.Z.); tianye@bmie.neu.edu.cn (Y.T.); mycao@nankai.edu.cn (M.C.)
† These authors contribute equally to this work.

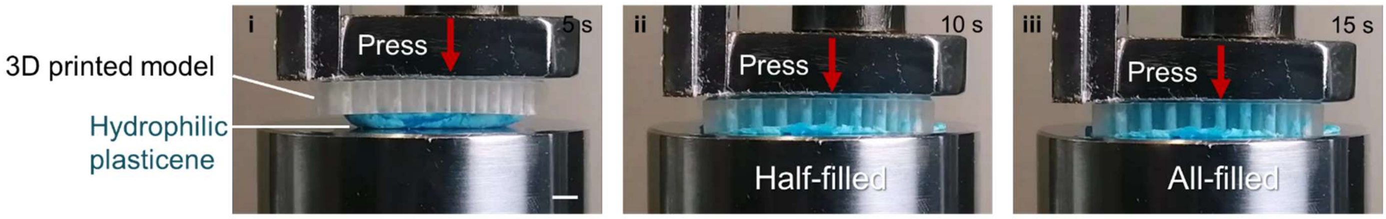

Figure S1. Shaping process of hydrophilic plasticene. The scale bar is 5 mm.

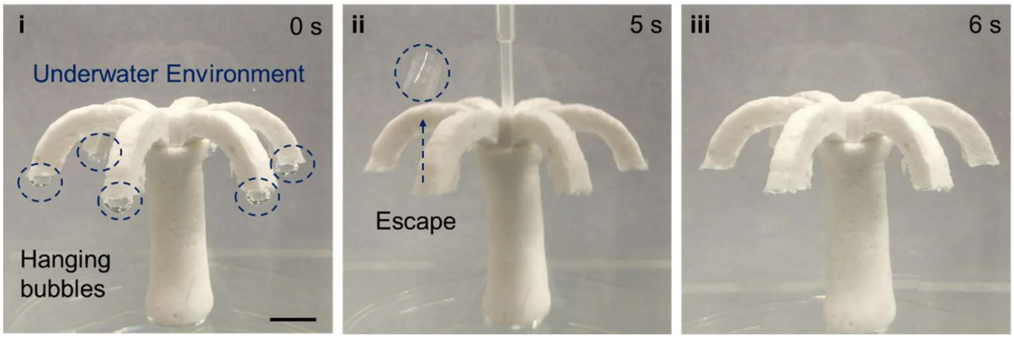

Figure S2. Gripping and releasing process of bubbles by tree-like hydrophilic plasticene in underwater environment. The scale bar is 1 cm.

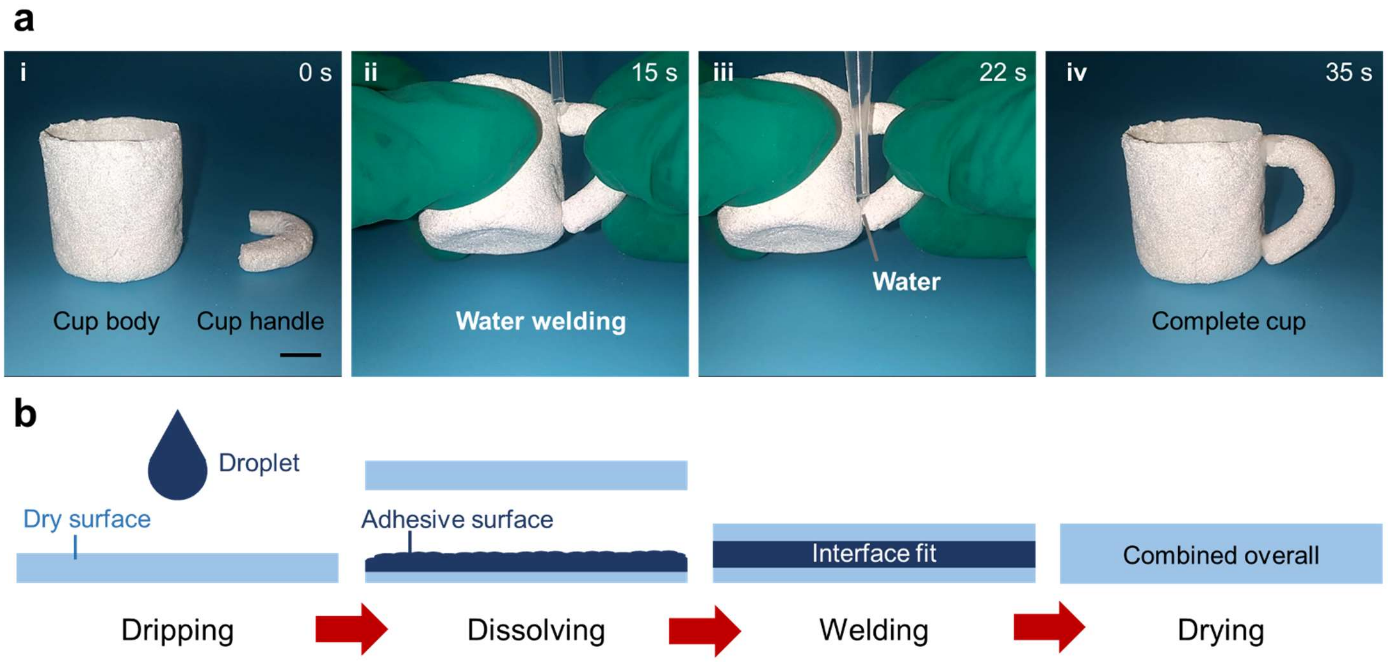

Figure S3. (a) Water welding process of hydrophilic plasticene. (i) The body and handle of the cup made of hydrophilic plasticene. (ii-iii) Dripping water at the connection. (iv) Get a complete cup. The scale bar is 1 cm. (b) Schematic diagram of water welding mechanism.

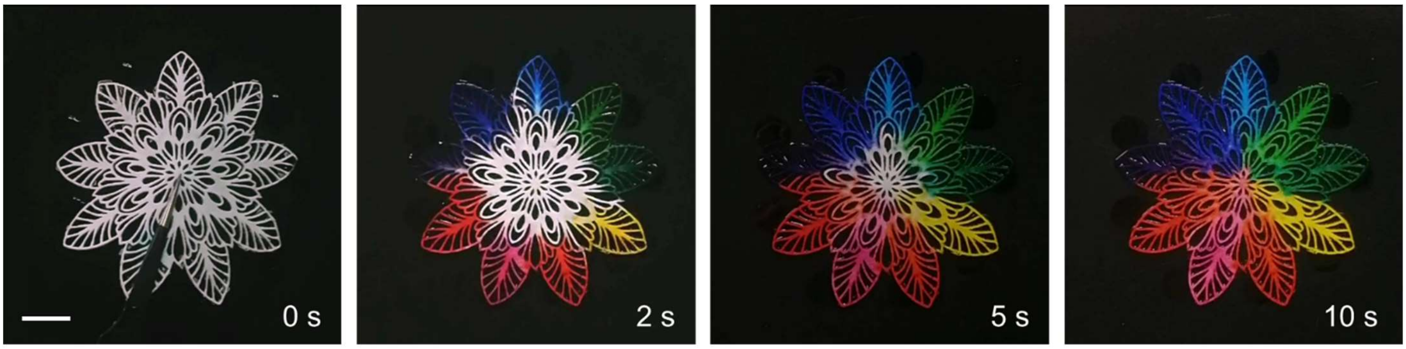

Figure S4. Colorful liquid transport process of flower-shaped hydrophilic plasticene. Nine colors of liquid transporting from the end of the petals to the center is finished in 10s. The scale bar is 1 cm.

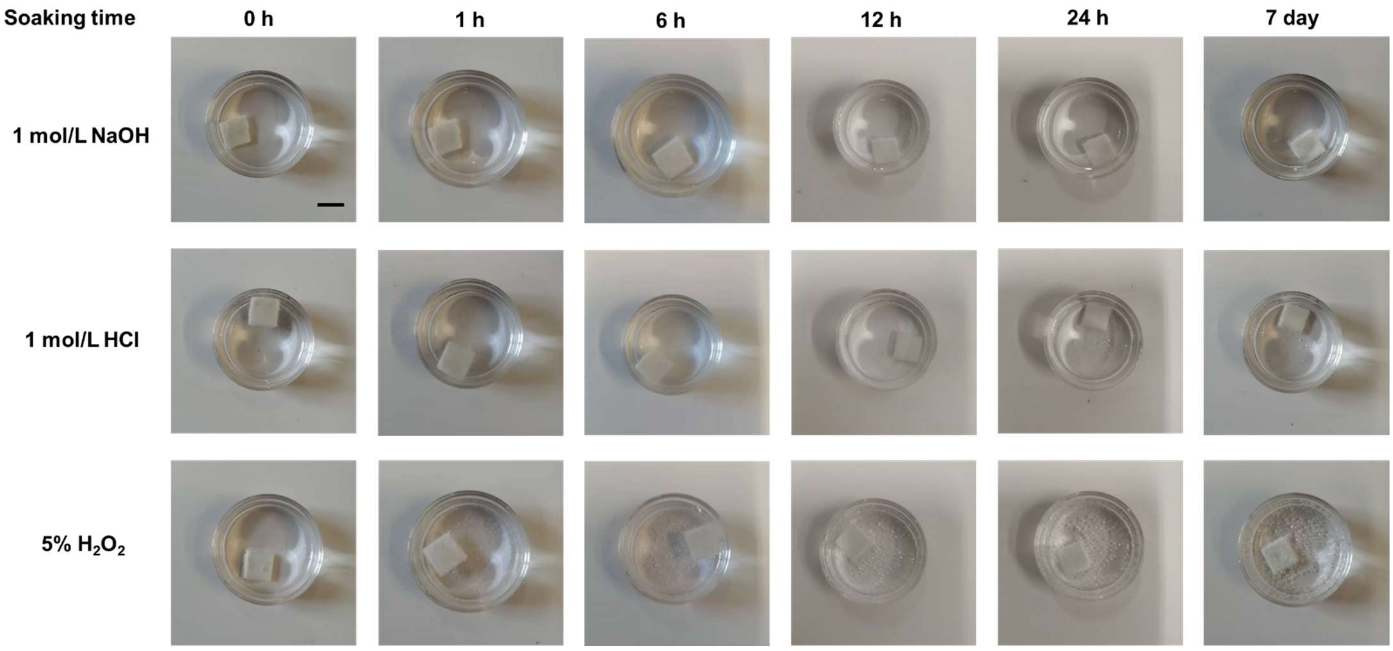

Figure S5. Alkali, acid and oxidation resistance of hydrophilic plasticene. The area of 1 cm^2, thickness of 2 mm sheet hydrophilic playdough were immersed in 1 mol/L NaOH, 1 mol/L HCl and 5% H_2O_2 solutions for 7 days, the morphology of the hydrophilic plasticene did not change significantly. The scale bar is 1 cm.

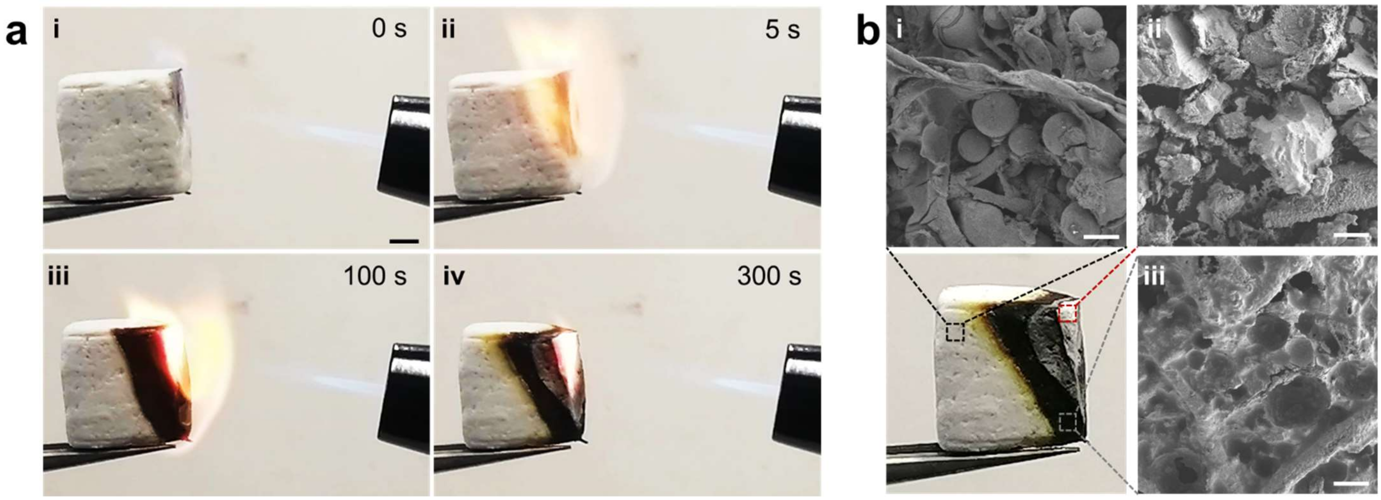

Figure S6. Superior fire retardancy of hydrophilic plasticene. **(a)** Snapshot taken at a fixed time of the experiment showing the effect of the hydrophilic plasticene cube (d=2cm) under butane gas combustion. The scale bar is 5 mm. **(b)** Photograph of the hydrophilic plasticene cube after six minutes of combustion under a butane flame. (i) SEM image of the portion of hydrophilic plasticene away from the flame, which still retains the intact structure of hydrophilic plasticene. (ii) SEM image of the portion of hydrophilic plasticene in direct contact with the flame, forming an inorganic powder that resists the heat of the flame. (iii) SEM image of the portion of hydrophilic plasticene not in direct contact with the flame, which is carbonized but still retains intact structure. The scale bar is 50 μm.

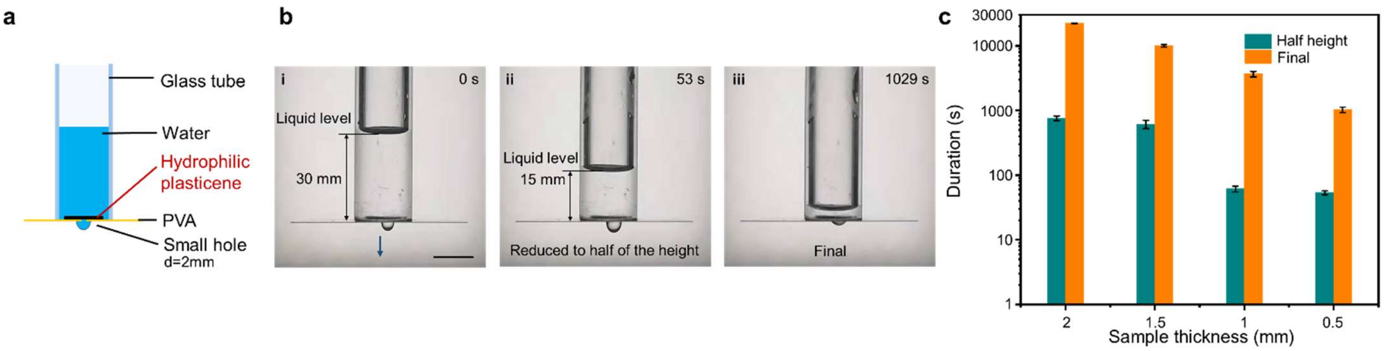

Figure S7. Water permeability of hydrophilic plasticene. **(a)** The water permeability measurement experimental setup with glass tube, hydrophilic plasticene (1 cm×1 cm) and PVA film. **(b)** Hydrophilic plasticene permeability process. (i) The height of water column at the starting stage is 30 mm. (ii) When the water column height is reduced to 15 mm, record the time. (iii) When the liquid level stops lowering for a long time, the timing is suspended. The scale bar is 1 cm. **(c)** Comparison of the time required for water to pass through samples of different thicknesses (2 mm, 1.5 mm, 1 mm,0.5 mm).

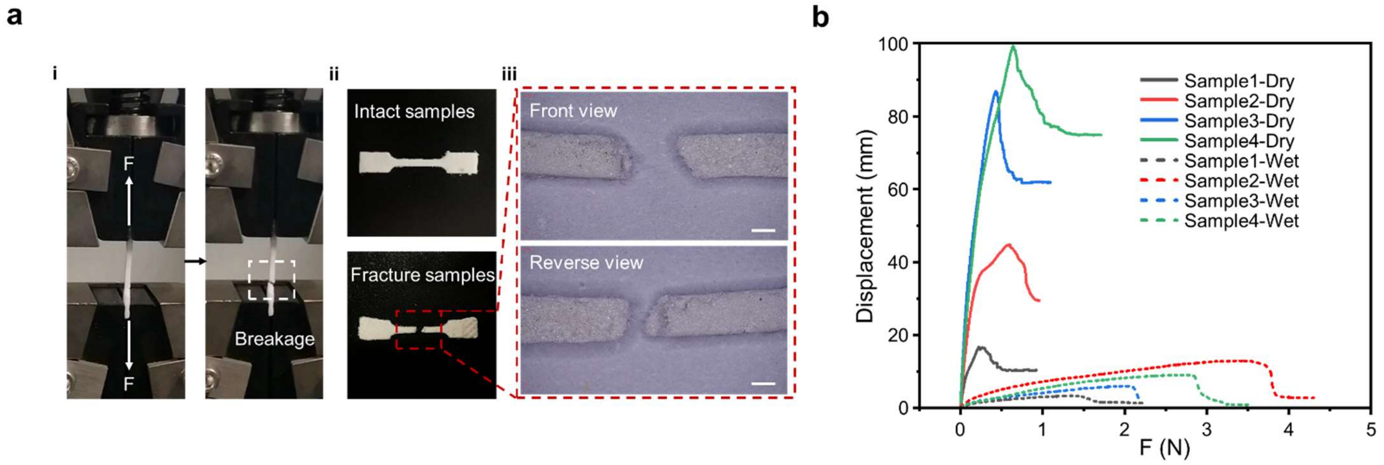

Figure S8. Mechanical properties of hydrophilic plasticene. **(a)** Mechanical properties performance test equipment and sample strips. (i) Hydrophilic plasticene sample stretching process. (ii) Comparison of intact and fracture samples. (iii) Comparison of the front and reverse sides of the sample breakage area. The scale bar is 1 mm. **(b)** Stress-strain curves of four samples in the dry and wet states. The ratios of Wood fiber, SiO_2 particle, and 5% sodium alginate solution in the sample strips were: Sample1=2：4：5, Sample2=2：4：5.5, Sample3=2：4：6, Sample4=2：4：6.5.

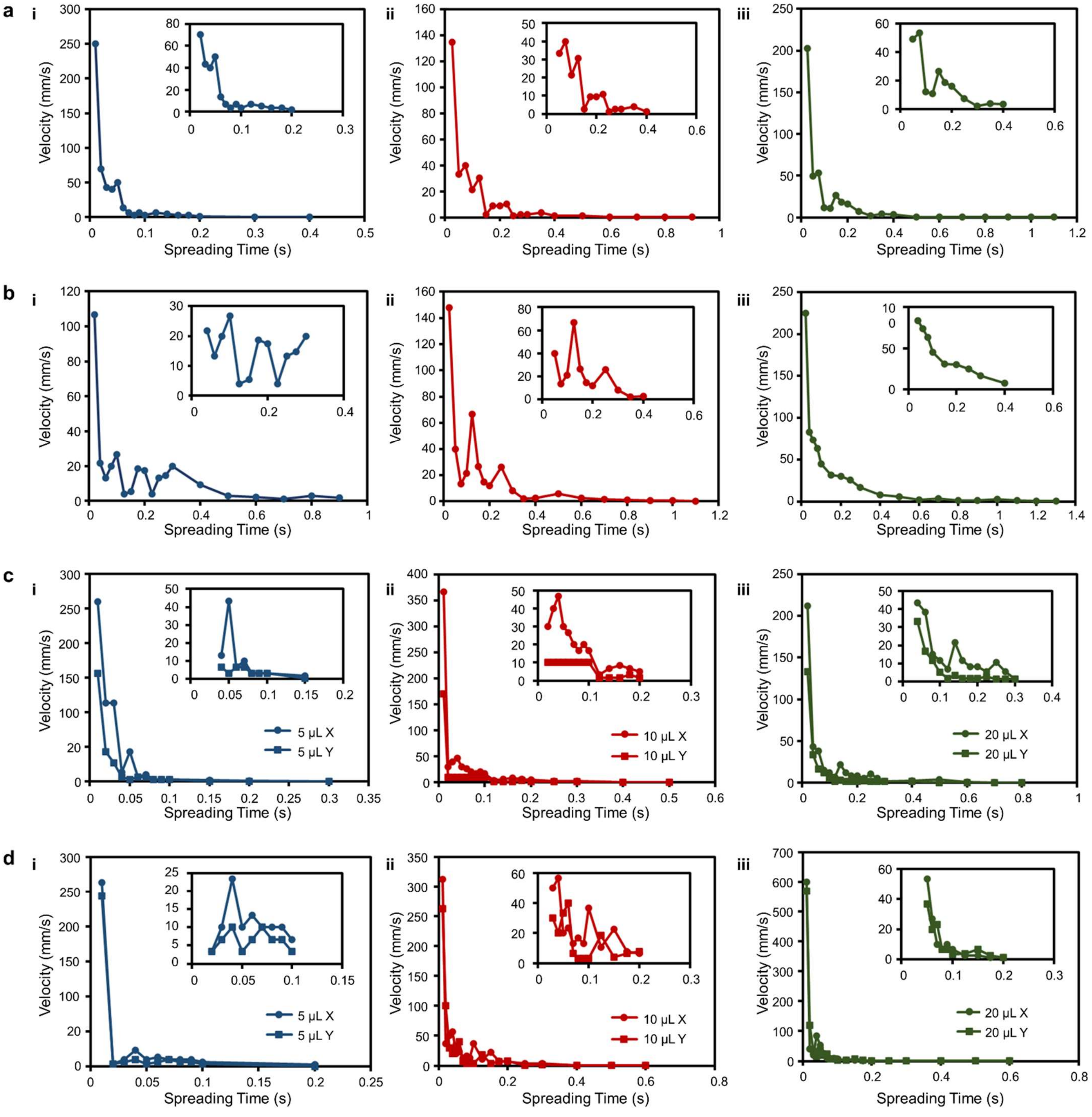

Figure S9. Relationship between spreading velocity and time of hydrophilic plasticene. **(a)** Thick plate. **(b)** Thin plate. **(c)** Stripe. **(d)** Square. (i) 5 μL droplet. (ii) 10 μL droplet. (iii) 20 μL droplet. The sawtooth tendency of the curves means that the spreading speed decreases and increases repeatedly.

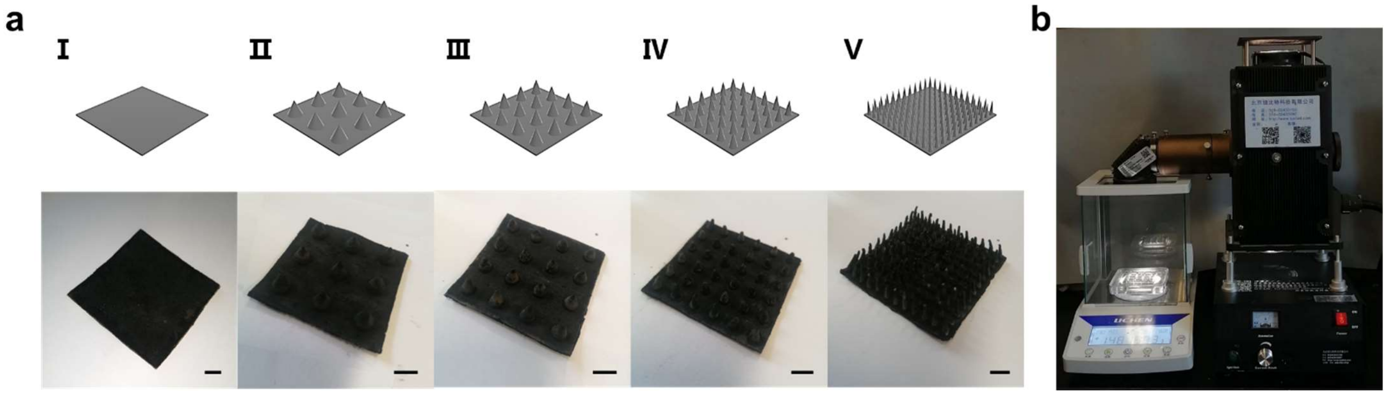

Figure S10. Photographs of the Solar evaporation ability test of hydrophilic plasticene in laboratory. **(a)** Five kinds of testing interface were selected. I is a 6 cm×6 cm square plate. II has 9 circular cones (base diameter=10 mm , height=10 mm) arranged as 3 × 3. III has 16 circular cones (base diameter=7.5 mm, height=10 mm) arranged as 4 × 4. IV has 36 circular cones (base diameter=5 mm, height=10 mm) arranged as 6 × 6. V has 144 circular cones (base diameter=2.5 mm, height=10 mm) arranged as 12 × 12. The scale bar is 1 cm. (b) Actual photos of the experimental setup.

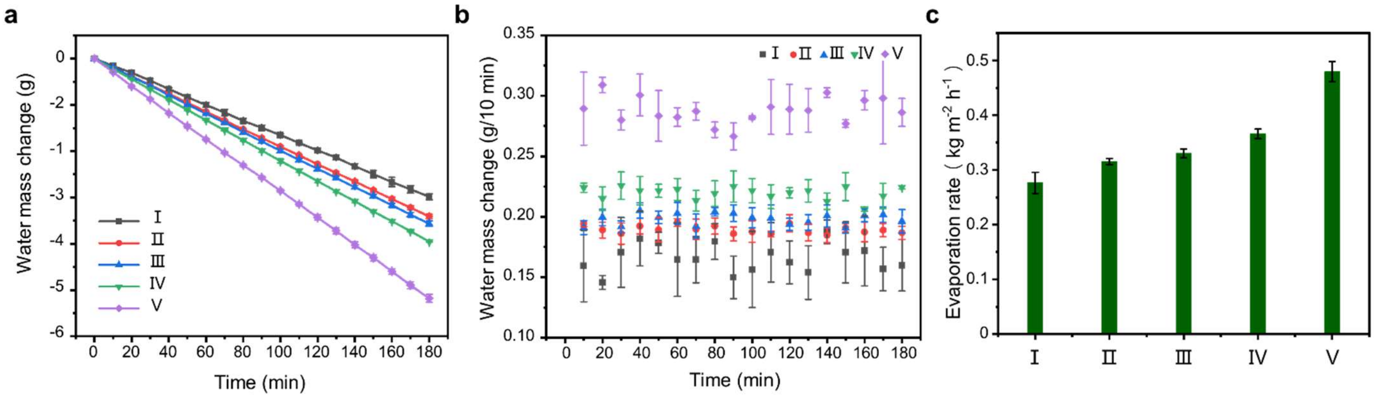

Figure S11. The in-lab evaporation performance of hydrophilic plasticene (without solar light). The water mass change **(a-b)**, evaporation rate **(c)** during 180 min indoor without solar light.

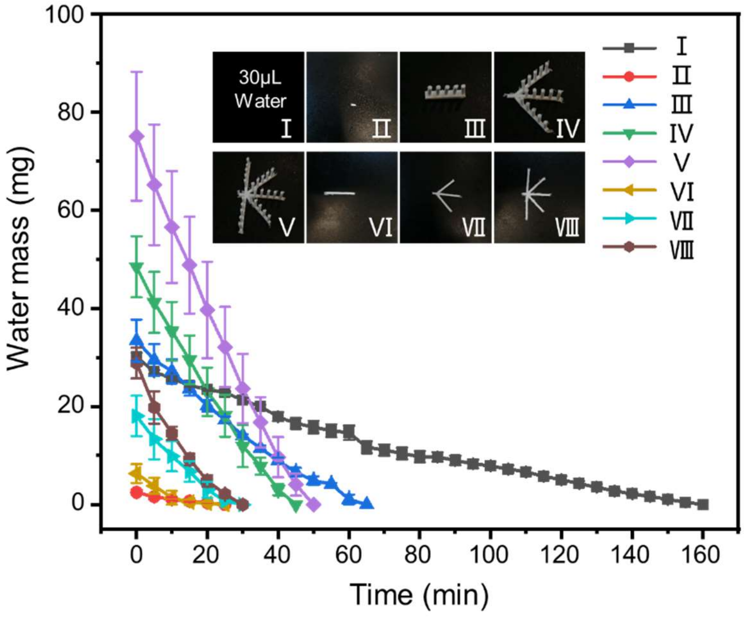

Figure S12. The in-lab evaporation performance of with or without cylindrical whole row of shaped hydrophilic plasticene and 30 μL water (without solar light).
